# Entrepreneurial learning in online communities

**DOI:** 10.1007/s11187-021-00502-8

**Published:** 2021-06-01

**Authors:** Peter Kalum Schou, Eliane Bucher, Matthias Waldkirch

**Affiliations:** 1 Department of Strategy and Management, Norwegian School of Economics, NHH, Helleveien 30, 5045 Bergen, Norway; 2 Department of Communication and Culture, Nordic Centre for Internet and Society, BI Norwegian Business School, Nydalsveien 37, 0484 Oslo, Norway; 3 Entrepreneurship & Family Firm Institute (EFFI), EBS Business School, EBS Universität für Wirtschaft und Recht, Rheingaustr. 1, 65375 Oestrich-Winkel, Germany

**Keywords:** Entrepreneurial learning, Online communities, Coactive vicarious learning, Social media, Content analysis, D83, L26, M13

## Abstract

**Abstract:**

New digital technologies possess the potential to transform entrepreneurial processes, such as how entrepreneurs pursue opportunities and access funding and how they learn. How entrepreneurs learn may be transformed as digital technologies provide new spaces for learning, such as online communities. Online communities can gather thousands of participants and provide entrepreneurs with new opportunities for learning that are not limited by time, space, or social class. Yet, we know little about how entrepreneurs take advantage of the new digital opportunities of learning. To remedy this, we studied a large online community of entrepreneurs on Reddit (r/startups), where we qualitatively analyzed the top-voted 100 threads from 2018 to 2019 (10,277 comments in total). By drawing on coactive vicarious learning, a theory that describes how learning is socially constructed through discursive interactions, we outline how entrepreneurial learning is socially constructed through conversations, which are taking place in different micro-learning contexts. Through identifying distinct *entrepreneurial learning conversations*, we build new theory on entrepreneurial learning in online communities. Our theorizing contributes to (1) the growing research on how entrepreneurial learning is socially constructed in communities, (2) the current debate on knowledge creation in online communities, and (3) the knowledge on how coactive vicarious learning unfolds in communities.

****Plain English Summary**:**

When entrepreneurs go online to learn, new research shows how online communities provide entrepreneurs with diverse learning spaces for developing ideas, learning new skills, and coping with the uncertainties of being an entrepreneur. Entrepreneurs increasingly use social media for doing business, but can they also use it to learn about doing business? In this article, we investigate this question by studying an online community of entrepreneurs on Reddit called r/startups, in which entrepreneurs exchange experiences and help each other with questions and issues. We show that entrepreneurial learning is taking place in five forms of learning conversations, which are situated in four learning contexts that differ from each other, from a *classroom* with a student–teacher dynamic, a *collab* space where entrepreneurs collect ideas and develop new skills and knowledge, a *club* context in which they challenge each other, and a *care* context in which they can bring their fears and uncertainties. Our findings show how entrepreneurship practitioners can make use of online communities, encouraging teaching and policy to pay more attention to how entrepreneurs work digitally.

## Introduction

Entrepreneurial learning is key to understanding why entrepreneurs succeed and how they may come back from failure. A significant stream of research has therefore investigated entrepreneurial learning (Breslin, 2019; Minniti & Bygrave, 2001; Toutain et al., 2017; Wang & Chugh, 2014). Studies have investigated a broad range of issues, encompassing how entrepreneurs learn from failure (Cope, 2011; Williams et al., 2020), how learning affects opportunity identification and exploitation (Corbett, 2005), how entrepreneurs learn from critical incidents (Cope & Watts, 2000), and how early success and easy access to legitimacy can hinder learning (Zuzul and Edmondson, 2017). Much of the literature on entrepreneurial learning has focused on studying learning as an *individual* activity (Toutain et al., 2017, p. 883).

While such individual-level approaches explain the cognitive processes of entrepreneurs (Corbett, 2005, 2007; Grégoire et al., 2011), they tend to neglect the role of social processes and communities in entrepreneurial learning (Hamilton, 2011; Toutain et al., 2017; Wang & Chugh, 2014). Only more recently studies have begun to analyze entrepreneurial learning as a socially embedded rather than individual process (Hamilton, 2011; Konopaski et al., 2015). This young stream of research investigates how entrepreneurs learn from others and how they learn as part of a community (Pittaway et al., 2015; Pugh et al., 2021; Zozimo et al., 2017), for example in student clubs and universities (Middleton et al., 2019; Pittaway et al., 2015) or family businesses (Hamilton, 2011; Konopaski et al., 2015).

Nevertheless, learning in online communities has not received much attention, as the literature has not yet engaged with digital opportunities for learning (Nambisan, 2017). Online communities provide new digital opportunities for entrepreneurial learning (Autio et al., 2013; Hwang, Singh & Argote, 2015; Leonardi, 2018; Nambisan, 2017), as they allow individuals to connect as part of a community that ignores boundaries of time, geographical distance, and even social hierarchy (Hwang et al., 2015). Investigating learning in online communities could enrich this nascent stream on entrepreneurial learning as a social process. Such investigation could also add to the growing interest in the digital aspects of entrepreneurship (Fischer & Reuber, 2011; Nambisan, 2017).

However, learning in online communities is driven to a higher degree by knowledge creation through communication (Faraj et al., 2011, 2016), as opposed to more traditional learning through observation (Hamilton, 2011; Konopaski et al., 2015). This makes classic learning theories, such as experiential learning (Kolb, 1984) and vicarious learning (Bandura, 1997), less suited in understanding learning processes taking place in online communities (Faraj et al., 2016; Myers, 2018). To grasp such specific form of social learning in online communities, we need to engage with how learning is discursively created (Myers, 2018). In this article, we, therefore, investigate entrepreneurial learning through the lens of coactive vicarious learning (Myers, 2018), a theory that outlines how learning is socially constructed as individuals share and discuss experiences.

Our paper aims to address the following question: *How do entrepreneurs learn as part of online communities*? To answer this question, we gathered data from the start-up community “r/startups” on Reddit, which counts over 380,000 users. Members use the online community as a space to discuss a wide array of entrepreneurial issues, such as marketing, team composition, or entrepreneurial exit strategies. The online community, therefore, provides a rich context to study entrepreneurial learning. In order to unveil learning dynamics and themes in the community (Levina & Vaast, 2015; McKenna et al., 2017), we inductively coded the top 100 upvoted, and thus most visible and active, threads from October 2018 to October 2019, which encompass 10,277 comments.

Our paper makes three contributions. We provide (1) insights into how entrepreneurs learn as part of an online community by outlining five conversations that pertain to key entrepreneurial concerns, such as personal narratives of success or failure, interpersonal conflicts with stakeholders, and business strategy. Building on these conversations, we argue that entrepreneurs learn not just through passive observation but also through active, discursive interactions, which we term *entrepreneurial learning conversations*. In doing so, we add to the young stream of research investigating entrepreneurial learning in communities as a social process (Pugh et al., 2021; Wang & Chugh, 2014; Zozimo et al., 2017). We further contribute to the (2) understanding of knowledge creation and sharing in online communities (Faraj et al., 2016) by showing how learning unfolds in diverse micro-learning contexts, thus providing new insights into the heterogeneity of digital learning. Finally, we add (3) empirical backing and nuance to Myers’ (2018) theory of coactive vicarious learning by applying it to the context of entrepreneurial learning in an online community.

The paper is structured as follows: First, we present an overview of the entrepreneurial learning literature. Second, we delve into the literature on vicarious learning on social media and we outline coactive vicarious learning theory (Myers, 2018). Third, we present our data and methods. Fourth, we outline our findings, and finally, we present a discussion and conclusion.

## Theoretical background

### From experiential to vicarious entrepreneurial learning

Entrepreneurial learning is a key ingredient in the entrepreneurial process as it allows entrepreneurs to improve their venture, product, and skills, and enables them to learn and possibly recover from failure (Cope, 2005, 2011). Entrepreneurial learning is also a key part of the effectuation process that drives venture creation (Sarasvathy, 2001). In framing and understanding entrepreneurial learning, research has drawn on various theories. Initially, experiential learning emerged as the dominant theory on individual learning of entrepreneurs (Wang & Chugh, 2014). Experiential learning theory defines learning as “the process whereby knowledge is created through the transformation of experience” (Kolb, 1984, p. 41). The theory outlines learning as a process of *taking in*, *interpreting*, *making sense of*, and *acting upon* information. Kolb (1984) imagines this as an idealized learning cycle where the learner continuously forms and reforms ideas and solutions via action, experience, and reflection. The theory of experiential learning has been applied in a multitude of contexts. For example, Corbett (2005) builds on it to differentiate how entrepreneurs identify and exploit opportunities, Politis and Gabrielsson (2009) employ it to understand how entrepreneurs learn from failure, and Pittaway and Cope (2007) draw from it to evaluate entrepreneurial education.

While experiential learning has brought many insights into the study of entrepreneurial learning, it has been criticized for insufficiently explaining how individuals learn with and from each other, since it mostly focuses on psychological processes and not on sociological ones (Holman et al., 1997; Kayes, 2002). Studies in entrepreneurial learning have, therefore, put a stronger focus on the social embeddedness of learning (Hamilton, 2011; Zozimo et al., 2017), building upon vicarious learning. In opposite to experiential learning, in vicarious learning, the learner draws from observing the behavior and its consequences from a model instead of learning from own performance outcomes (Gioia & Manz, 1985). Examples of such research are Zozimo et al.’s (2017) study of how entrepreneurs learn from observing role models, Pittaway et al.’s (2015) work on learning in student clubs, and Hamilton’s (2011) study of how family businesses can function as communities of practice, where entrepreneurs learn from their family how to run a business. This version of vicarious learning theory has also been used to understand how employees in organizations gain knowledge from online communities (Hwang et al., 2015; Kane, 2017; Leonardi, 2014, 2017). For example, Leonardi (2014) analyzes how an online community serves as a knowledge-sharing device, which allows employees to share knowledge more easily through a virtual space than a physical space.

Although vicarious learning has provided novel insights into how entrepreneurs learn from each other and how individuals can gain knowledge from engaging in online communities, it has recently come under scrutiny from scholars pointing out serious limitations. Myers (2018, 2020) argues that vicarious learning simplifies learning into stable, unidirectional learning relationships between an expert and a novice. In this learning relationship, the expert simply transfers their knowledge to the recipient, the novice. It represents a troublesome simplification because individuals do not learn through “consuming” others’ experience, but through a “give and take” process, in which experience is analyzed and discussed (Myers, 2018, 2020). Therefore, classic vicarious learning theory limits our understanding of learning in communities (Myers, 2020) and, in particular, in online communities, in which learning is facilitated by active knowledge creation through conversations (Faraj et al., 2016; Myers et al., 2018). Classic vicarious learning theory is simply not able to conceptualize learning as socially constructed through conversations (Baker et al., 2005; Faraj et al., 2016; Kolb & Kolb, 2005; Myers, 2018), making it ineffective in understanding the social interactions in online communities that create knowledge and facilitate learning (Faraj et al., 2016). In order to investigate how entrepreneurs learn in online communities, we draw on coactive vicarious learning (Myers, 2018), which focuses on learning as a social process that takes place through conversations. Hereby, we are taking a sociological approach nested in symbolic interactionism (Blumer, 1986; Myers, 2018) in contrast to previous research on entrepreneurial learning favoring a cognitive focus (Grégoire et al., 2011).

### Framing entrepreneurial learning through coactive vicarious learning theory

Myers (2018) shifts vicarious learning towards a more relational process that occurs coactively. Myers (2018, p. 613f) defines coactive vicarious learning as “a discursive learning process where individuals (i.e., a model and learner) intentionally share and jointly process a model’s work experience(s) in interpersonal interactions to coconstruct an emergent, situated understanding of the experience(s).” By taking into account the exchanges, relationships, and knowledge sharing that define online communities (Faraj et al., 2016), Myers’ theory is well fitting to capture and understand entrepreneurial learning processes in online communities. For example, Myers et al. (2018) use the theory to understand how surgeons use social media to learn as it allows them “to discursively react to one another’s ideas and coconstruct a more robust, detailed understanding of their experiences” (Myers et al., 2018, p. 235).

Myers (2018) outlines three key discursive elements that constitute coactive vicarious learning interactions: experience, analysis, and support. Coactive vicarious learning unfolds as individuals share and gain access to a greater number of *experiences*, which function as a basis for reflection and the development of knowledge. For instance, through storytelling, groups can share, compare, and build on each other’s experiences, allowing them to create new shared knowledge in the process. Coactive vicarious learning further unfolds through the *analysis* of shared and co-constructed experiences, which allows members to “evaluate, reinterpret, or compare their emerging understanding of the experience” (Myers, 2018, p. 618). Such analysis can unfold through probing, asking for clarification or criticism. Last, *support* by the community enhances an individual’s learning by creating safety and allowing them to develop beliefs and relationships in the community. For instance, friendship communities foster the transmission of more tacit and intimate knowledge. These three elements shape the learning context in critical ways; for example, a context rich in support may facilitate deeper conversations about failure and learning from such critical events (Myers, 2018, p. 618).

The coactive vicarious learning perspective is promising because it could help improve our understanding of entrepreneurial learning as a socially embedded process situated in communities, a perspective that has received little attention in the literature (Hamilton, 2011; Toutain et al, 2017; Wang & Chugh, 2014). Consequently, we seek to explore how experience, analysis, and support—as key elements of co-active vicarious learning—manifest in online conversations among entrepreneurs and how these conversations differ in dynamics and style.

## Methodology

### Research context

In order to gain insight into how entrepreneurs use online communities as a space for knowledge creation, we collected data from an online community of entrepreneurs (“r/startups”) on Reddit. The community consists of 382,000 members with several hundred commenters being online at any given time. The community space is public and openly accessible—even to users without a registered account—and there is no expectation of privacy among the members (Sugiura et al., 2017). Commenters are anonymous and identify with a chosen online handle. The community is dedicated to “discussing startup problems and solutions.” As emphasized by the moderators, the purpose of the community is to “support others, educate others, inspire others and foster authentic relationships.”

### Data collection

In our sampling, we strived to select threads that had the best chance to answer our research question (Lincoln & Guba, 1985). To this end, we compared daily threads (all threads) as well as “top-rated” threads (most “upvoted” threads). While both types of threads contained similar themes, the daily threads usually had much less activity than the top threads. Following a purposeful sampling logic (Lincoln & Guba, 1985; Patton, 2002), we collected the top 100 threads within a span of 12 months (October 2018–October 2019) with all their respective comments, resulting in a total corpus of 10,277 comments. The longitudinal data was scraped via the Python Reddit API wrapper (PRAW). Reddit defines the success of community threads based on the number of “upvotes” received by community members. The selected top-rated threads received between 794 and 113 upvotes, and they encompass between 625 and 10 comments each. In gathering successful threads over a longer period, we preclude short-term spikes or trends in the conversation from distorting the overall sample and provide often-read and representative threads (Levina & Vaast, 2015; McKenna et al., 2017).

### Data analysis

**Fig. 1:**
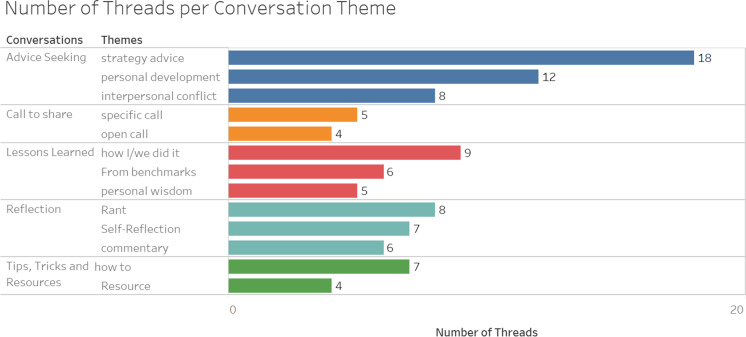
Thread categories

To analyze the data, we relied on a mix of context-driven inductive and theory-driven structured coding processes (Glaser & Strauss, 1967; Miles, Huberman, & Saldaña, 2014). Such ways of analyzing data from online communities have gained prominence in recent years (Levina & Vaast, 2015; McKenna et al, 2017; Vaast et al, 2017). Our data analysis encompasses four steps. First, in order to familiarize ourselves with the online community environment, all researchers observed the online community daily between September 2019 and October 2019, creating an inventory of recurring conversation dynamics, themes, language, and community-specific idiosyncrasies. In a second step, we collected, categorized, and clustered the top 100 threads—consisting of post title and post text—of the past 12 months according to the type of learning conversation.^1^ In the descriptive analysis, we built clusters of conversations according to observed conversation dynamics (e.g., advice-seeking, lessons learned, call to share, and providing resources) as well as conversation content (e.g., self-reflection, strategy advice, and interpersonal conflict). The initial clustering revealed five distinct conversation dynamics: lessons learned, advice-seeking, call to share, reflections, as well as tips, tricks, and resources^2^ (see Fig. 1). In these two first steps, we sought to structure our data following an inductive approach (Glaser & Strauss, 1967).

In our third step, we sought to build theory on how entrepreneurs interact within the online community to construct knowledge. To this end, we used Myers’ (2018, pp. 617–618) analytical framework and described and compared each of the identified conversation clusters with respect to the three discursive elements of coactive vicarious learning—experience, analysis, and support. We compared the conversations with respect to (1) how experiences were shared within the community and as a back-and-forth between posters and commenters, (2) how experiences were collectively analyzed, as well as (3) how community members socially and emotionally supported each other during the knowledge building.

The analysis of online conversations further revealed two specific patterns: learning conversations across categories differed not just with respect to conversation dynamics and discursive elements but also with respect to their *emotionality* (tone and language of conversations range from technical to emotional) and their *level of abstraction* (framing of conversations ranges from concrete to abstract). Emotionality effectively deepens the discursive element of support (technical vs. emotional support) while the level of abstraction primarily adds nuance to the discursive element of analysis (concrete vs. abstract frame of analysis). To shed further light on these two patterns, we decided to include an additional fourth step of data analysis by coding all 100 threads for *emotionality* (technical = 1 vs. emotional = 2) as well as *level of abstraction* (low = 1, medium = 2, high = 3). While the level of abstraction inductively emerges as a dimension from the data, our understanding of the dimension is aligned with Trope and Liberman’s (2010) work on construal levels. Through this last step or analysis, we identified four “micro-learning” spaces, which differed in terms of emotionality and abstraction, thereby indicating heterogeneous learning contexts. To secure the credibility of our findings (Pratt et al., 2020), we applied investigator triangulation (Denzin, 1970). All authors independently coded all threads and subsequently discussed diverging cases (11 in total) until a consensus was reached.

## Empirical findings: entrepreneurial learning in online communities

Our empirical analysis reveals that the entrepreneurs in the online community engage in five distinct entrepreneurial learning conversations: *lessons learned*, *advice-seeking*, *reflection*, *call to share*, as well as *tips, tricks, and resources*. We present the entrepreneurial learning conversations below (see Table 1 for an overview and Tables 2, 3, 4, 5, and 6 in the Appendix for representative data^3^).

**Table 1:** Analytical framework of entrepreneurial learning conversations and coactive vicarious learning (CVL)

Learning conversations	Themes	Discursive elements of CVL		
		Sharing experiences	Analyzing experiences	Offering social and emotional support
Lessons learned	▪ Personal narratives of success or failure ▪ Importing third-party experiences and benchmarks ▪ Small “nuggets” of personal wisdom Typical post title: “My First Startup Failed. Here’s Everything I Learned From It”	▪ Mentor/students dynamic ▪ Narratives combine experiences and personal conclusions ▪ Conversation focuses on original post	▪ Asking for clarification and additional details ▪ Contrasting initial lesson with own experiences ▪ Collecting cases which prove or disprove the lesson learned	▪ Support focuses on brief affirmations and compliments ▪ Emotional support mostly limited to lessons derived from failure
Advice-seeking	▪ Interpersonal conflicts with internal and external stakeholders ▪ Personal career development ▪ Advice on general business strategies Typical post title: “I hate talking to customers. Any tips to get over it?”	▪ Student/mentors dynamic ▪ Narratives surround an open issue or conundrum ▪ Rich conversation surrounding original post and comments	▪ Interpreting and paraphrasing original issue ▪ Proposing and discussing step-by-step solutions ▪ Advice-givers bolster credibility through position and tenure ▪ Deep engagement with issues presented	▪ Support focuses on increasing self-efficacy through actionable advice ▪ Some cynicism in reply towards “incapable” advice seekers and advice givers
Reflection	▪ Opinionated commentary on the start-up landscape ▪ Voicing personal frustration ▪ Emotional self-reflection Typical post title: “Three years in and feeling burned out”	▪ Friends/supporters dynamic ▪ Sharing of personal and intimate narratives and ideas ▪ Rich and extensive conversation surrounding original post and comments	▪ High level of community engagement ▪ Comparing and contrasting original narrative with own experiences and insights ▪ Offering psychological advice	▪ Support focuses on personal and emotional validation ▪ Community provides a safe space for personal reflection ▪ Peer validation and comfort especially for “rants” and “self-reflections”
Call to share	▪ Open call to pitch individual start-ups ▪ Specific call to share knowledge within the community Typical post title: “Share your startup—May 2019” “Startup CEOs: What is your average workday like?”	▪ Moderator/participants dynamic ▪ Additive sharing of stand-alone narratives ▪ “Sender-oriented” conversation with limited interaction among commenters	▪ Collecting and contrasting experiences ▪ Lack of evaluation or interpretation due to “additive” nature of conversation	▪ Support is not a predominant discursive element within this category ▪ Emotional support limited to shared negative experiences
Tips, tricks, and resources	▪ Sharing process knowledge and tips (how-to) ▪ Sharing tools and resources Typical post title: “The Ultimate Term Sheet Guide—all terms and clauses explained”	▪ Coach-students dynamic ▪ OP shares tips, tricks, and resources based on books or based on skill, e.g., programming ▪ In more rare examples, OP shares tips, tricks, and resources based on own experience	▪ Evaluating success or usefulness of resource ▪ Aggregating related resources ▪ Improving and complementing provided tool sets	▪ Support focuses on brief notes of gratitude for shared resources ▪ Some support towards self-efficacy

### Entrepreneurial learning conversations in online communities

#### Lessons learned

The first identified cluster of conversations encompasses “lessons learned.” In this learning conversation, one user generally starts a conversation by sharing a specific personal experience of success or failure, often followed by a more abstract analysis of “how they did it” as well as their personal conclusion. In some cases, lessons learned are not first-person experiences but derived from industrial benchmarks, such as Twitter or LinkedIn. A typical prompt within the lessons learned category contains both storytelling and abstract analysis—often both these elements are already present in the thread title: “My First Startup Failed [storytelling]. Here’s Everything I Learned From It [analysis].” Conversations following the “lessons learned” schema often evolve into a mentor/student dynamic with the initial poster remaining the primary focus of the conversation.

By way of analyzing the originally provided “lesson,” other commenters often ask for clarification, additional details, or background stories so as to better apply the lesson learned to their own experiences. For example, replying to a particularly bold “lesson learned,” one user asks: “Quick question: What gave you the confidence that you could pull this off in the first place?” Some commenters also provide similar accounts, reiterating the original post, or point towards differing experiences of their own, adding to it. Posters of “lessons learned” often remain engaged in the conversation and take an active part in offering advice and emotional support to commenters. Some posters end their initial “lesson learned” with a call to ask questions and engage in conversation: “Feel free to ask questions, or talk about your own experiences, I’m always open to receiving advice.” Peer reactions and engagement in the lessons learned conversations depend on the tone and content of the initial post. Lessons based on personal failures often prompt brief and sympathetic utterances of support such as “sorry for your failures but look at […] all you learned.” Other peer support is more social than emotional, often consisting of a brief “good post, thanks for sharing,” providing affirmation to the poster. Within the community, it is frowned upon to come across overly self-confident or bragging. Thus, even when users share their achievements, they take care to signal their grounding in reality. For example, one user prefaces their spectacular success story with a note of modesty: “I dont mean to be braggadocious because I know we still have so much to learn and so much farther to go.”

#### Advice-seeking

The second cluster consists of conversations surrounding “advice-seeking.” Here, the initial prompt is an open-ended problem or question to which the poster has not conceptualized a solution yet. Themes of these conversations encompass questions about strategizing, about personal development, and about how to approach and solve interpersonal conflicts. A typical post in this category starts with a personal narrative detailing the conflict or problem and with a call for advice or feedback. Some prompts are concrete and surrounding an imminent problem, such as: “[My] coder cheated: Can I trust him anymore?” or “I hate talking to customers. Any tips to get over it?” Others are broader and look for more general insight. One poster asks the community if they have “any advice for a young CEO.” The dynamic of advice-seeking conversations is one between a student and a community of coaches or mentors and provides a more collective learning approach.

Most analysis takes place first by (re-)interpreting or paraphrasing the posters key conundrum before putting forward a solution, such as: “[if the coder shows signs of dishonesty,] make sure you hire a third party developer to check for any backdoors in the codebase before you fire him.” Advice is often structured in actionable and sometimes numbered steps for the original poster to follow. The conversation within the advice-seeking category is generally rich as commenters do not only reply to the original issue but also give feedback to other comments. Here, there is a culture of emphasizing one’s own professional track record and experience to bolster one’s competence as an advice giver (e.g., “I’m an Industrial Engineer who’s programmed for almost 20 years”). Commenters sometimes extend the advice-giving paradigm to include feedback or criticism towards the advice seeker—especially if they feel that they contributed in part to their current predicament: “‘Should I trust this super sketchy guy I let keep working for me after he already screwed me over in a major way?’ I don’t expect stellar decision making from [original poster].”

#### Reflection

The third category of conversations encompasses reflections within the safe space of the community. Unlike the advice-seeking threads which center on actionable guidance, the reflections contain open-ended narratives without calls for explicit advice. On the one hand, reflections take on the form of intimate and emotional self-reflections or frustrated “rants” or venting. A typical self-reflection post comes from a start-up founder who is “three years in and [is] feeling burned out.” In the post, they detail their emotional state and seek to “hopefully open the floor to other entrepreneurs that feel alike.” On the other hand, reflections can be more general commentaries on the current start-up landscape, containing threads with titles such as “Startup Culture On College Campuses Is Broken,” where an entrepreneur comments on the skewed incentives for entrepreneurial activity, which invite a “culture of starting a startup for fame, money, and power rather than for solving a problem.”

Reflections usually prompt particularly high community engagement with commenters taking on the role of a friend or even personal coach, offering compassionate advice or sharing similar experiences and reflections in order to validate—and oftentimes console—the initial poster: “Don’t lose sight now! It may take some time, but your next breakthrough will come.” The significant share of reflection threads tying into mental health, burn out, and other instances of personal crisis brings forth a new discursive style focused primarily on the element of support. Within the reflection paradigm, instances of cynicism or criticism voiced towards the initial poster are exceptionally rare. Instead, commenters signal their readiness to engage in further discussion by creating a friendly and “safe” discursive space. For instance, commenters often conclude their posts with a note inviting further conversation: “Hope this helps my friend—I’m always happy to talk anything through so feel free to shoot me a [direct message].”

#### Call to share

The fourth cluster concerns threads initiated by a “call to share.” These conversations start as either open or specific calls for member contributions. Open calls recur monthly and are prompted by a forum moderator. Members are encouraged to present or pitch key facts of their start-up. A typical reply to an open call provides *URL*, *location*, *idea pitch*, *information about what they are looking for in the community*, as well as possible *discounts* that they offer to fellow community members. In the more specific calls to share, members ask the community as a whole or a defined subgroup within the community to share knowledge about a specific aspect of entrepreneurial life: “Startup CEOs: What is your average workday like.”

The calls to share follow a moderator-participant dynamic with the moderator providing the initial prompt and the participants engaging in multiple distributed micro-conversations (potentially one conversation per pitch). While the open calls usually receive a very large number of replies, only the most “upvoted” pitches actually generate conversations in which participants ask questions, offer to be test users, or provide feedback. Conversations following the open call to share paradigm are often technical expert conversations with low levels of emotionality and occasional questions or challenging interjections such as “Tbh I don’t see a market need for this at all. […] Just giving you my honest feedback.” Emotional and social support mainly encompasses short encouraging notes such as “nice, good luck!” Specific calls to share, on the other hand, are much richer in community engagement, and they provide a broader umbrella for discussions.

#### Tips, tricks, and resources

The final conversation cluster identified was “tips, tricks and resources.” Here, initial prompts provide either process guidance, explaining how to approach and tackle a specific task, or they would share a tool, repository, or resource that would be useful to fellow entrepreneurs. Unlike the lessons learned—where posts often entail generalized strategic or personal advice—the tips and resources shared in this category are very actionable and implementation oriented, focusing on a narrow and concrete application area.

A typical post in this category tackles the question of “how to price your product in a way that communicates quality.” Another post provides “The Ultimate Term Sheet Guide—all terms and clauses explained.” These highly specific posts tend to be rather long, often exceeding 1000 words and offering detailed explanations with sources, links, tables, or other appendices. While conversations within this paradigm often garner many upvotes—indicating appreciation by the community—they seldom receive many comments. When members do decide to leave comments, they either convey their gratitude to the original poster for their work in compiling and sharing resources and they contribute additional resources of their own: “Agree with all of this my dude. [I would] also recommend this eBook on anything working on SaaS pricing.” A final characteristic of the kind of cluster is the low level of emotionality and emotional support.

Overall, the conversations we outline above illustrate that the way learning takes place in an online community differs along Myers’ (2018) three elements of experience, analysis, and support (see Table 1). Following the idea of heterogeneity among learning conversations, we found that they are not uniform across the community but take place in *micro-learning contexts* that are marked by varying degrees of emotionality and levels of abstraction. In the next section, we outline these contexts.

### Micro-learning contexts in online communities

**Fig. 2:**
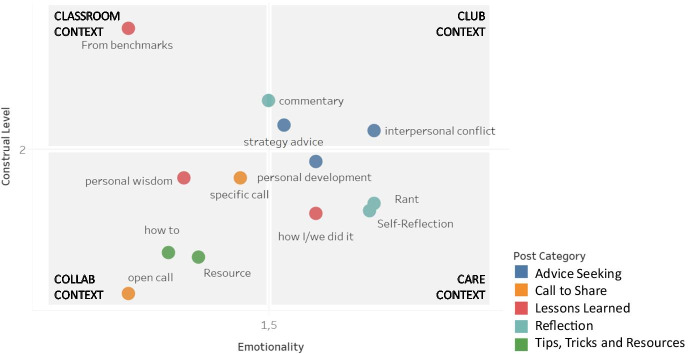
Micro-learning contexts

Our results further indicate that entrepreneurial learning within the online community takes place against the backdrop of four specific micro-learning contexts (see Fig. 2), marked by varying degrees of emotionality and levels of abstraction (construal levels). We found that the identified learning themes unfold in (1) a *classroom* context (high abstraction, low emotion), (2) a *collab* context (low abstraction, low emotion), (3) a *club* context (high abstraction, high emotion), as well as (4) a *care* context (low abstraction, high emotion).

The first micro-learning context can be compared to a *classroom space* in which learners evaluate, interpret, and compare abstract knowledge and ideas, often in a rather technical manner. The classroom learning context encompasses primarily threads from the lessons learned paradigm, especially threads that analyze successes or failures of “best-in-class” benchmarks or high-level methods or theories. In the classroom context, learning generally unfolds in an abstract and scholarly manner, in which “teachers” (original posters) often introduce or summarize knowledge from external sources. On the side of the “students” (community members), the classroom space functions as an open forum with low participation thresholds. However, there seem to be rather high participation thresholds on the side of the “teacher” whose original input post is expected to fulfill almost scientific standards, often spanning upwards of 1000 words. For example, after presenting an overview of the lean start-up philosophy, one author is repeatedly asked to provide a source for their post and to “acknowledge the real writer of this work,” to which they promptly supply a reference. The tone and language used in the classroom context are often technical and focused on rendering the introduced knowledge as accessible and “digestible” as possible. As an example, community members often break down the original post and provide a short summary to their fellow “students” to facilitate the discussion of the overarching ideas.

The second micro-learning context works akin to a collaborative or “collab-space” where entrepreneurs meet to discuss and build concrete and often skill-based knowledge by exchanging resources, guidelines, and action-oriented lessons that they have gathered during their entrepreneurial tenure. Tone and language are collegial but not overly emotional; members of collab spaces often discuss on eye level without a discernable hierarchy and without a strong focus on the initial poster. Most posts are met with a brief thank you and a follow-up question or with additional knowledge or experiences. Collab spaces span several different entrepreneurial learning conversations such as lessons learned, calls to share, as well as tips, tricks, and resources. While the collab space is an active environment for technical and even “nerdy” debates, there are no discernible entry barriers and entrepreneurs of varying levels of experience and maturity feel comfortable sharing their own experience and contributing with comments or questions. When asked why an experienced user with “20 years bouncing between being a consultant, a [venture capitalist] and an entrepreneur” would take the time to share their insight with their less experienced counterparts, they reply: “I’m a big believer in spontaneous serendipity […] If I can be that […] for someone, then I’m thrilled. This start-up world is hard. We need as many good people helping each other as we can get.”

The third micro-learning context works like a society or a club space. In this space, “members” evaluate, interpret, and compare medium- to high-level learnings or questions which are often derived from personal experiences such as entrepreneurial success or failure. As such, the evolving discussions can be emotional and even personal in nature. The club space is primarily a background for advice-seeking and higher-level reflections. In particular, members use club spaces to discuss interpersonal or strategy issues that they have personally encountered. The club space is marked by rather high participation thresholds and in-community hierarchies with members regularly offering their “credentials” in the form of experience, tenure, or job title as a precursor to their contributions. Unlike in the collab space, answers offered in the club space are often questioned and challenged by other members. One user who seeks advice on coping with their “lying CEO” receives multiple and sometimes conflicting pieces of advice that are fiercely discussed within the community. While some advise the user to “leave [the company] ASAP,” others interject that “it is a bit soon to advise [the original poster] to leave the company.” While some say to inform investors immediately, others warn not to contact investors under any circumstances before the [original poster] has “lawyered up.”

The final micro-learning context can be characterized as a highly informal and personal care space where members take over roles of confidantes or friends. Here, the issues discussed are tangible and concrete, encompassing everyday events or states of mind. Care spaces span multiple learning conversations, such as reflections—often expressed as short “rants” about current personal predicaments—or personal lessons learned. Conversations within care spaces are marked by a highly emotional language and tone. Here, emotionality is often conveyed through the use of emotional verbs (love, like, hate, etc.), adjectives (happy, sad, afraid, desperate, depressed, etc.), or nouns (stress, burnout, anxiety) as well as the use of punctuation to create “emoticons” such as smiley faces or multiple exclamation marks. Further emotionality cues are derived from personal terms of endearment such as “friend,” “buddy,” or “brother”/ “sister.” Care spaces are “no-threshold” zones where all users are welcome and validated—both as original contributors and as commenters. As such, care spaces are especially inviting environments for “cathartic rants” as one user puts it. Unlike in the classroom or collab space where the quality of content and generalizability is a central concern, the care space allows for purely introspective and individual reflection: “It’s like burnt rubber in your chest. […]I don’t want to do this. I hate calling people. I hate sales. I hate bothering people. […] I hate the way my voice trembles on the phone. I hate the way my ribcage feels frozen when I’m waiting for people to pick up the phone.” Replies and comments within the care space all follow a pattern of non-judgment and validation where users either echo the sentiment (“Bro…I know that feeling”) or offer advice (“it’s time to hire an assistant to do the cold calling for you”) or a word of courage or support (“Gorgeous rant! I hope it was cathartic for you. It was for me.”).

As our findings indicate that entrepreneurial learning occurs both in different conversations that encompass various topics and in different micro-learning contexts, which present different ways of coactive vicarious learning. Therefore, the way a particular conversation unfolds may be markedly different, depending on the micro-learning context it takes place in.

## Discussion and conclusion

Our findings offer insight into entrepreneurial learning in online communities by providing an inventory of *entrepreneurial learning conversations* that entrepreneurs engage in—highlighting how entrepreneurs learn through sharing and analyzing experiences as well as through social support—and by mapping out different *micro-learning contexts* in which conversations of different emotionality and level of abstraction take place.

### Online communities as catalysts for higher-level entrepreneurial learning

Building on coactive vicarious learning, our findings provide novel insights into the nascent research on how entrepreneurs learn as part of communities in general (e.g.Hamilton, 2011; Zozimo et al., 2017) and online communities in particular (Faraj et al., 2016). We extend the literature on learning in communities by highlighting how engaging in online communities may enhance critical self-reflection and higher-level learning (e.g., Cope, 2003, 2005) and how online communities may differ in their requirements for participation compared to other communities (Hamilton, 2011; Konopaski et al., 2015).

First, our findings suggest that entrepreneurs in online communities learn from sharing and analyzing experiences of critical events such as failures (e.g., a venture does not scale), disruptions (e.g., co-founder leaves venture), or conflicts (e.g., suspected employee misconduct). Such learning is particularly present in conversations surrounding “advice-seeking,” “lessons learned,” and “reflections.” This closely corresponds to Cope (2003, p. 445) who emphasizes that “non-routine events represent a key entrepreneurial learning mechanism” as they spark *critical self-reflection* that leads to distinctive forms of fundamental “higher-level learning” (as opposed to routine events which stimulate more incremental lower-level learning). Our findings highlight that online communities may be a powerful catalyst for such critical reflection and higher-level learning. On the one hand, an entrepreneur who experiences a discontinuous event might find an online community where their own experience and behavior are mirrored, commented on, questioned, and exposed by other entrepreneurs to be particularly conducive for critical reflection and higher-level learning. On the other hand, an entrepreneur who has not (yet) experienced a specific discontinuous event could still be peripherally exposed to such fundamental learning experiences and thus vicariously observe critical self-reflection and higher-level learning, which may help in navigating such critical events in the future. Thus, online communities, as anonymous conversation spaces, may provide particularly immediate, intimate, and unfiltered insights into how others have experienced, analyzed, and dealt with discontinuous events.

By shedding light on entrepreneurial learning in the socially embedded context of online communities, our research contributes further to Cope’s (2005, p. 385) call for “more research on distinctive forms of learning that arise from the entrepreneur’s engagement in social relationships.” While Cope (2005) suggests looking towards family and domestic partners as “sounding boards” for entrepreneurial learning, we propose to complement the perspective with specialized online communities, such as Reddit. Against this background, we encourage future research to scrutinize higher vs. lower level learning in online communities, as well as to investigate how and across which conversations critical self-reflection and higher-level learning occur in online communities (Cope, 2003). To understand such processual phenomena, we encourage future research to engage in longitudinal netnographies (Kozinets, 2010) or other processual methods.

Second, whereas previous research illustrates how entrepreneurs learn through deep engagement and socialization into a community, such as a family business (Hamilton, 2011; Konopaski et al., 2015), our findings indicate that in most cases, socialization plays a secondary role in online communities such as Reddit. Online community members can seamlessly access and exit conversations and thereby engage in coactive vicarious learning, without having to invest extensive time legitimizing their presence. It does not mean that online communities are complete without legitimacy requirements. For instance, in conversations surrounding the “lessons learned” paradigm, entrepreneurs generally have to signal credentials, such as tenure, knowledge, or experience of critical events. Future research may engage more with access thresholds and legitimacy requirements across different communities and how they may affect learning dynamics.

### Online communities as heterogeneous micro-learning contexts

Our finding that the online community consists of four separate micro-learning contexts extends our knowledge on online communities as social spaces for different conversations. Levina and Arriaga (2014, p. 477) argue that online communities should be seen as a “social space engaging agents in producing, evaluating, and consuming content online that is held together by a shared interest and a set of power relations among agents sharing this interest.” We extend such theorization by providing new insights indicating that online communities do not consist of a singular social space, but multiple, heterogeneous spaces—the micro-learning contexts—in which social interactions differ. For example, conversations among entrepreneurs differ in terms of the emotionality shown. This is important for two reasons. First, in highlighting these different spaces, we provide an alternative to the network-based analysis of online communities (e.g., Johnson et al., 2015). Our model allows researchers to distinguish an online community by how interactions create certain spaces, rather than by who is connected. Thereby, the model can facilitate research into the knowledge creation in online communities, which is inhibited by network analysis (Faraj et al., 2016).

Second, our findings on micro-learning contexts connect to the growing recognition of the importance of the social and relational context around learning activities (Politis et al., 2019). For example, Pugh et al. (2021) show how universities can foster reflective and interactive cultures where participants actively engage to share and analyze experiences, which helps build up local entrepreneurial ecosystems. Our findings of how such contexts are created can help researchers understand how individual entrepreneurial learning can transform into joint learning, which benefits communities rather than just individuals (Pugh et al., 2021). As we outline, the contexts can take very supportive and caring dimensions, as seen in the case of “care-spaces” where entrepreneurs can share failure and receive support. These insights may provide an impetus for future research into where entrepreneurs turn to cope with failure (Cope, 2011; Simmons et al., 2014).

### The social construction of entrepreneurial learning

Although there has been a recent surge in research analyzing entrepreneurial learning as a socially embedded process (Hamilton, 2011; Pittaway et al., 2015; Pugh et al., 2021; Zozimo et al., 2017), Toutain et al. (2017, p. 883) argue that the entrepreneurial learning literature still lacks “a better understanding of the interactionist processes that play a role in the construction of social learning.” Our study improves understanding of how entrepreneurial learning is socially constructed in the following ways. First, we illustrate that entrepreneurial learning conversations in an online community can cover a broad range of topics that vary in emotionality and complexity. Thus, the interactionist processes that play a role in the construction of social learning should be understood as different processes. They vary in topic and, importantly, in emotionality, complexity, and abstraction. The implication of such difference is that learning in a community is defined not just by the type of community but also by topic. For example, a family business is a specific type of community (Hamilton, 2011), yet learning in a family business is also defined by topic. Learning about continuity in a family business is likely an emotional topic, due to the socioemotional wealth present in the business (Konopaski et al., 2015). Learning about marketing in the same family business may not be emotional, but perhaps an issue characterized by complexity. Therefore, scholars should not just differentiate entrepreneurial learning by what community it takes place in, but what the learning effort is about. In doing so, scholars may open for more fine-grained research on entrepreneurial learning in communities.

Second, we outline how different learning dynamics unfold in different conversations in the online community. In our findings, the “lessons learned” and the “advice-seeking” conversations have what we term a mentor-student dynamic. The mentor-student dynamic differs from the similar vicarious learning dynamics identified in previous research by having a more active student role (c.f. Hamilton, 2011; Konopaski et al., 2015; Zozimo et al., 2017). In the “lessons learned” conversations, the students actively engage with the mentor and ask questions and contrast with own experience, and sometimes they seek to disprove the mentor’s claims. In the “advice-seeking” conversations, the students start the conversation by actively seeking advice. Previous research has tended to portray students as passive following classic vicarious learning theory, e.g., a daughter hanging out in business premises (Konopaski et al., 2015, p. 359). Our findings indicate that students play a more active role and that learning is not necessarily unidirectional; students can challenge the mentor, provide analysis of the experience shared, and also contrast it with their own experience. Moreover, we also portray a different learning dynamic. In our “reflection” conversations, the dynamic is more between equal friends and supporters and not an unequal relation between a mentor and a student. The finding contrasts with previous research, which assumes power differences between a mentor and a student (e.g., Zozimo et al., 2017). In sum, our findings indicate that entrepreneurial learning in online communities is nuanced; it covers different levels of emotionality and complexity, and it also includes different dynamics.

### Theoretical contributions

By integrating research on entrepreneurial learning (Hamilton, 2011; Pittaway et al., 2015; Pugh et al., 2021; Wang & Chugh, 2014) and coactive vicarious learning (Myers, 2018) in the empirical context of online communities (Faraj et al., 2016), we provide novel insights into how entrepreneurial learning processes unfold, making three contributions. *First*, we extend the young stream of research investigating entrepreneurial learning in communities (Hamilton, 2011; Pittaway et al., 2015; Pugh et al., 2021) by providing novel insights into how entrepreneurs learn as part of online communities. In contrast to previous research, which has investigated how entrepreneurs learn through personal engagement in small, local communities, e.g., a daughter observing her father running the family business (Konopaski et al., 2015), we outline how entrepreneurs learn through *entrepreneurial learning conversations* in a global online community. In these conversations, entrepreneurial learning is socially constructed as entrepreneurs share experiences and insights, analyze them, debate them, and provide emotional support in sensitive matters. By drawing on coactive vicarious learning theory, we illustrate how entrepreneurial learning is achieved through active, discursive interactions in contrast to classic vicarious learning that limits entrepreneurial learning to passive observation (Myers, 2018, 2020). Our findings indicate that these conversations can help entrepreneurs both in mundane matters and with higher level learning as particular conversations may trigger critical reflection (Cope, 2003). Our focus on conversational learning is well grounded in the learning literature (c.f. Baker et al., 2005; Kolb & Kolb, 2005; Myers, 2018) but has not yet been applied to the entrepreneurial learning literature. Our paper may therefore be a steppingstone for future research to fully realize the potential of studying interactionist processes that create entrepreneurial learning (Toutain et al., 2017).

*Second*, we contribute to the growing interest in how vicarious learning takes place in online communities (Faraj et al., 2011, 2016) and how knowledge is constructed and shared (Faraj et al., 2016). Building on Myers’ (2018) theory to analyze knowledge sharing and learning as conversations, we uncover how the emotional depth and construal level of the discussions (Trope & Liberman, 2010) create micro-learning contexts, namely *classroom context*, *collab context*, *club context*, and *care context* that influence learning among entrepreneurs, thus underlining the importance of online communities as learning spaces for entrepreneurs. Thereby, we extend knowledge on learning spaces in online communities (Faraj et al., 2016). By enriching the understanding of the heterogeneity of digital learning spaces, our model of multiple micro-learning contexts further improves understanding of online communities as social spaces, which consist of different social practices (Levina & Arriaga, 2014).

*Third*, we translate Myers’ (2018) organizational theory of coactive vicarious learning into the context of online communities and provide empirical evidence of coactive vicarious learning. We extend his theory by nuancing the support and analysis dimensions, adding new insights into how emotionality and construal levels construct micro-learning contexts. These findings add to understanding the importance of the learning context, as highlighted in both experiential learning theory (Kolb & Kolb, 2005) and vicarious learning theory (Myers, 2018). Future research could further investigate the different micro-learning contexts that arise in communities. For example, studies of venture incubators may find different levels of successful learning due to different micro-learning contexts, thus shifting the focus further from “what” to “how” entrepreneurs learn.

### Practical implications

Our study has several practical implications. First, our study emphasizes the growing importance for entrepreneurs to engage in online communities. As our findings indicate that taking an active part in learning conversations provides entrepreneurs with an important impetus for learning and can be especially beneficial for entrepreneurs with a lack of access to traditional entrepreneurship communities. Similarly, entrepreneurs, who may be isolated without close colleagues, can find help online. Second, our study might be of interest to entrepreneurship educators outside the traditional higher education context. Engagement with entrepreneurs outside a higher-education setting is underexplored, yet a clear opportunity for entrepreneurship education (Nabi et al., 2017). Online communities provide an exciting opportunity to engage with entrepreneurs from various contexts and to “democratize” entrepreneurship education. Third, our findings indicate the importance of creating various learning contexts to teach the various shades of entrepreneurship, from more tool-based approaches toward understanding team dynamics. Our findings suggest that different topics might thrive better in a specific micro-learning context. We thus encourage educators and organizers of entrepreneurship events to focus on the learning context they create. In light of the COVID-19 pandemic and crisis, these implications are especially important. Entrepreneurs are facing an unprecedented crisis in which they require all the help they can get. As we revisit the online community on Reddit, we note that how to deal with the COVID-19 crisis has become a primary focus of entrepreneurs. Thus, online communities may become a contingent resource during times of crisis, in which quick reactions and support matter.

### Limitations

As an inductive study on a new phenomenon, our study is not without limitations. First, we rely on data from one online community on Reddit. Although it is a large community with more than 380,000 members, future research should investigate the extent to which our findings are transferable across different online communities. Second, while our analysis considers how conversations play out, we do not investigate specifically how different trends or types of entrepreneurial learning conversations change over time, which opens up future research opportunities for more process-driven studies. Third, by taking a social constructionist approach through which we focus on how learning is created through conversations, we are limiting our focus on the individual, cognitive side of learning, e.g., how entrepreneurs transform experience into knowledge. For future research, it may be prudent to pursue an experiential learning perspective (Kolb, 1984) to complement our coactive vicarious perspective. Fourth, while we provide insights into entrepreneurial learning conversations, we cannot show specific learning outcomes. This is a result of our methodology and choice of an inductive research question; we thus encourage future research to test learning outcomes of entrepreneurs in online communities. Last, while our analysis is based upon the conversations among entrepreneurs, in line with other research on online communities (McKenna et al., 2017), we encourage future research to additionally collect other types of qualitative data, such as interviews or diary studies, to further triangulate the findings.

## Conclusion

Understanding the way that entrepreneurs learn has increasingly received attention, because it represents an important aspect of the entrepreneurial process. In this article, we show how entrepreneurial learning unfolds through specific entrepreneurial learning conversations across micro-learning contexts of online communities. Thereby, we challenge and extend current thinking on both entrepreneurial learning as well as online communities. On the one hand, we show how online community spaces can act as uniquely fruitful catalysts for entrepreneurial learning conversations as they provide a *multi-faceted* and *low-threshold* environment for entrepreneurs to engage in and render conversations accessible both in real time and in retrospect (c.f. Hwang et al., 2015). This will be especially important considering the increasing societal importance of online community spaces as avenues for public discourse and as repositories for (entrepreneurial) stories, strategies, and resources. On the other hand, challenging the notion of online communities as homogeneous spaces for learning, our article is a call for future research to engage with how the nature and topography of online communities affect and mold learning processes of entrepreneurs (Nambisan, 2017). Based on our exploration of entrepreneurial learning within a specific online community, we urge entrepreneurship researchers to draw on new theories, concepts, and methodologies to further map and understand the “architecture of participation” that online communities offer to entrepreneurs (Nambisan, 2017).

## Appendix

**Table 2:** Themes and sample quotes of advice-seeking

Post category	Post subcategory	Sample post title	Sample quote
Advice-seeking	Interpersonal conflict	Co-founder is harassing me	[My co-founder has] started harassing me by making inappropriate comments […] Being a small startup, there’s no HR to go to for help […]Should I just walk away from this startup and try to start over on my own?
		Our “CEO” secretly invested the last 30% of our funding in crypto…	“[Our CEO] was the only person with access to the accounts and [over] half our funding has been squandered at this point.”
		CEO is lying to potential clients and investors about our number of paid users. And it’s in writing	Our CEO is lying to potential clients/investors about our number of paid users. […] Is he violating any laws or is this just a shady business practice? Looking for some feedback or advice
		Coder cheated: Can I trust him anymore?	Need your advice on this…I found out [the developer] installed a cheat application to manipulate the screen tracking tool. […] I don’t trust him […] How do you approach a scenario like that?
	Personal development	Is it foolish to start a startup if you have almost no savings?	I am about to graduate from college and work on this full-time. […] My personal savings are close to nothing. My parents are quite poor and won’t be able to support me financially. […] Do you think it is a foolish and a dangerous choice? Or is there a potential that this may work?
		I hate talking to customers. Any tips to get over it?	I’m an introvert and a perfectionist and it’s impacting my businesses. […]Any tips for how to engage or get feedback without feeling embarrassed or stupid?
		Leaving my cushy corporate job for a startup?	“Currently stuck weighing the two options in my head. […] Not sure what to do here. Any input or personal experience with something similar is welcome!”
		How do I put my failed startup on my resume?	After six months I have $0 revenue, 0 employees, and I’m having a hard time figuring out how to summarize my experience in a couple of bullet points. Especially since it feels like I have accomplished nothing. Any help would be appreciated. Thanks
	Strategy advice	How do you successfully manage a remote team?	What tools, strategies, advice do you recommend for successful remote teams? Best hiring practices? Is there anything you should watch out for or avoid?
		Why do engineers keep declining our offer after talking to their wives?	As soon as [our candidates] talked to their wives they told us they had to decline. So my question is, why do the wives keep telling our potential engineers no? Is it because we are both women?
		I’m a newly-anointed CEO of a small company with 900 K ARR. I own ~ 10%. How much should I pay myself?	What is a fair total number to pay myself? * Knowing that I am incentivized by performance-based pay, how should it be structured?
		If “You don’t need to go to school to become an entrepreneur” where do you learn business finance?	Sure you can hire accountants, lawyers, and bring in friends but how do you know that your lawyer is giving you good advice and that your accountant isn’t taking advantage of you if you truly know nothing about such things?

**Table 3:** Themes and sample quotes of call to share

Post category	Post subcategory	Sample post title	Sample quote
Call to share	Open call	Share your startup—January 2019	Tell us about your startup! […]What stage are you in? How many employees or founders? Are you looking for anything? (Feedback/Hiring/Investment)
		Share your startup—April 2019	Tell us about your startup! […]What stage are you in? How many employees or founders? Are you looking for anything? (Feedback/Hiring/Investment)
	Specific call	What are some habits of highly UNsuccessful founders? (Anti-Patterns)	There’s a lot of prescriptive successful founder patterns, but not a lot written about anti-patterns. […]Please be specific and constructive
		Teach us a lesson. Share your failed business stories	Hey, we love hearing success stories of how people create startups based on their brilliant ideas and then skyrocket. […] But I believe those who failed have much more to tell and teach us! Share main reasons why your ex-businesses failed
		Startup CEOs: What is your average workday like?	I am an apprising startup owner i am interested in what the day to day life of a startup CEO is like. What do you do during your days? Where do you work at? What kind of hours do you work?
		What emerging technology or trend is flying under everyone’s radar?	Obviously, everyone is still focused on AI/ML (AKA statistics), VR, Augmented Reality, etc., but I wonder if there isn’t something a bit more obvious staring us in the face

**Table 4:** Themes and sample quotes of lessons learned

Post category	Post subcategory	Sample post title	Sample quote
Lessons learned	From benchmarks	How Slack Got Their First Users?	“There are four famous growth hacks behind the Slack’s success: Blue Ocean strategy, minimal features focus, freemium business model, fear of social isolation hook.”
		How LinkedIn Got Their First Users?	Growth doubled when Hoffman decided to invite only successful friends and connections, recognizing that cultivating an aspirational brand was crucial to drive mainstream adoption. The local critical mass that breeds both user loyalty and word of mouth (virality) was met within the following four months
	How I/we did it	My First Startup Failed. Here’s Everything I Learned From It	I created my first startup when I was still in high school. I made mistakes, lost more money than I dare to mention, and ultimately had a huge public failure. […]Feel free to ask questions, or talk about your own experiences, I’m always open to receiving advice
		The Five Biggest Things I’ve Learned From Building A Start Up	Saying “take your ego out of things, don’t take things personally” is a lot easier than actually doing it. As much as it hurt to be told that whoever I’d just pitched to didn’t care, it motivated me 10x more. I became immune to the fear of rejection
		Year 2 update now 10 K / month	“It has been about a year since I last posted our update on our company. […] I found a great co-founder/friend and we were off to the design, iterate, repeat. […] You cannot do everything yourself so you need to build your team to help with the stuff you don’t really like or have time to do.”
		3 days: 12 cups of coffee: 24 k lines of code & a MVP is born	“Just want to share my story of the past three days with you guys and maybe provide inspiration to someone who just doesn’t think they can do it.”
		From a sucky accounting job to doing $1.4 million dollars a year with my mobile app 4 years after launch! (And I can’t write a single line of code). How I did it, and what’s next!	Pull up a chair family! I’m going to peel back the layers to show that this stuff is actually doable. And while we’re not making 10′s of millions like some other apps yet, I think we have a real path to get there. […]This is a post on how I did it
	Personal wisdom	The team you start with is not the one you scale with	I have decided not to join any early stage startups as I want to be the “scale up” employee instead of the “start up” employee. Looking at the odds, they are the ones who get Max ROI
		[UPDATE] Lessons learnt from a startup event	Networking is very important. One guy in my team already has a startup and has pitched a lot of times before investors. He got the chance to pitch in front of them through networking. […] One big entrepreneur actually hooked up with him on LinkedIn

**Table 5:** Themes and sample quotes of reflection

Post category	Post subcategory	Sample post title	Sample quote
Reflection	Commentary	Most startups are actually marketing problems	I have come to the conclusion that most startups face marketing as their core problem. Even if you have the best product in the world that people do actually want, it’s pretty useless unless they know about it
		Anyone else feel like there’s a cultish feel and overuse of the book The Lean Startup?	Its always MVP, its always this way of testing the brand, product, etc., and I think its way overused, especially by people who have not built companies
		Snap should just hit back at FB and copy them	Snap is the only non-FB property that has a big enough network already that it could threaten FB if it had the right product. 5. Couldn’t Snap just build out a competing product to FB?
	Rant	A rant about why we’re failing	I know what the problem is. I know why we’re not successful. We really should be calling hundreds of people a day. We should be trying all of our wild ideas. We should be gathering information as fast as possible and iterating. I should be doing this. […]But I’m scared shitless of all of it. I hate that I have enough goodwill built at the company that I’m getting away with it. I hate that people describe me as “productive” when it’s all glossy shit that in the long-term amounts to nothing. It’s eating away at my soul
		100% of 0 is nothing	I’ve started **(and failed)** multiple businesses and it is 100% my fault, every time. I recently came up with an idea and threw it around to a few people I knew. My good friend/ old roommate was more excited about it than I was
		Stop Wasting your Time Asking for Likes/Shares for a Startup Competition	The number of entrepreneurs I see wasting time asking for likes/shares for a worthless startup contest/competitions is outstanding. […] These contests are a distraction from your objectives, which are growing your business
	Self-reflection	3 Years in and Feeling Burned Out	The problem is that after 3 years, I feel burned. I do not have the same passion for the position and the project as I had before, I’m starting to arrive late at the office and leave earlier, it takes me longer to complete tasks and overall procrastination has started to become a problem. […] Thank you for listening to me
		Founder Depression	I should be happy, I should be focused, I should be really engaged in the business. […] My cofounder has decided to move on (after 3.5 years) and I’m not sure what to do. My wife wants to see other people. She says that I’ve changed ever since I started this business
		Have I wasted 10 years of my life?	I was caught up in the zeitgeist of starting a startup but 10 years ago at the age of 37 I took the leap and started my own company. […] If I stayed a vp where I was I would most likely be on board level now with a significantly better spread of wealth. […] Don’t know what I want from this post but I am struggling to find my motivation

**Table 6:** Themes and sample quotes of tips, tricks, and resources

Post category	Post subcategory	Sample post title	Sample quote
Tips, tricks, and resources	How to	How to Launch a Lean Startup	Lean startups can save significantly on overheads and are more likely to deliver a product that fits a real market need. To implement this philosophy, it’s important to consider several aspects that may run counter to established business norms, but deliver real results
		Why User LTV (Lifetime Value) Should Be a Key Priority for Your Startup	User Lifetime Value (LTV) is the measure of a user’s worth over a period of time. […] This metrics will help you optimize your acquisition strategy, targeting users that give-back. So let’s dive deeper in it…
		How to price your product in a way that communicates quality	One of the most common mistakes tech entrepreneurs (especially first-time ones) make is to **charge too little**. […] The “right” price is often the one that makes us uncomfortable. As a rule of thumb, I always price my products 2X or 3X what I’m “comfortable” charging for. Then I force myself to sell at that price point
	Resource	A humble glossary of mistreated terminologies	I thought that maybe it would be nice to share a few definitions, and what they really mean. Feel free to add yours
		The Ultimate Term Sheet Guide—all terms and clauses explained	We’ve written a long guide on term sheets at Salesflare, which, if you read it, can make the difference between a good and a bad VC deal. Because with term sheets, the devil is in the details… Enjoy!

## Footnotes

1 Myers (2018) uses the term discourse. We avoid this term due to its multiple meanings in different literatures. Instead, we use the simpler term conversation with inspiration from Baker et al. (2005).

2 For an overview of themes and sample quotes, see Appendix.

3 See Appendix.
